# microRNAs: Critical Players during Helminth Infections

**DOI:** 10.3390/microorganisms11010061

**Published:** 2022-12-25

**Authors:** Maura Rojas-Pirela, Diego Andrade-Alviárez, Wilfredo Quiñones, Maria Verónica Rojas, Christian Castillo, Ana Liempi, Lisvaneth Medina, Jesus Guerrero-Muñoz, Alejandro Fernández-Moya, Yessica Andreina Ortega, Sebastián Araneda, Juan Diego Maya, Ulrike Kemmerling

**Affiliations:** 1 Instituto de Ciencias Biomédicas, Facultad de Medicina, Universidad de Chile, Santiago 8380453, Chile; 2 Laboratorio de Enzimología de Parásitos, Departamento de Biología, Facultad de Ciencias, Universidad de Los Andes, Mérida 5101, Venezuela; 3 Instituto de Biología, Facultad de Ciencias, Pontificia Universidad Católica de Valparaiso, Valparaiso 2373223, Chile; 4 Núcleo de Investigación Aplicada en Ciencias Veterinarias y Agronómicas, Facultad de Medicina Veterinaria y Agronomía, Universidad de Las Américas, Santiago 8370040, Chile

**Keywords:** miRNAs, *Schistosoma*, *Fasciola*, *Brugia*, immunomodulation

## Abstract

microRNAs (miRNAs) are a group of small non-coding RNAs that regulate gene expression post-transcriptionally through their interaction with the 3′ untranslated regions (3′ UTR) of target mRNAs, affecting their stability and/or translation. Therefore, miRNAs regulate biological processes such as signal transduction, cell death, autophagy, metabolism, development, cellular proliferation, and differentiation. Dysregulated expression of microRNAs is associated with infectious diseases, where miRNAs modulate important aspects of the parasite–host interaction. Helminths are parasitic worms that cause various neglected tropical diseases affecting millions worldwide. These parasites have sophisticated mechanisms that give them a surprising immunomodulatory capacity favoring parasite persistence and establishment of infection. In this review, we analyze miRNAs in infections caused by helminths, emphasizing their role in immune regulation and its implication in diagnosis, prognosis, and the development of therapeutic strategies.

## 1. Introduction

Helminths are complex organisms that comprise approximately three hundred thousand species that can be either free-living or parasitic [1]. They include some taxonomic groups, such as trematodes (flukes), cestodes (tapeworms), and nematodes (roundworms), associated with infections in animals and humans [2].

Infections with *Schistosoma mansoni (S. mansoni)*, *Fasciola hepatica (F. hepatica)*, and *Brugia malayi (B. malayi)* species are among the most common helminth infections in humans [3], causing nutritional, physical, and cognitive impairment [4,5]. Moreover, some of these helminth infections can also promote cancer development, being considered biological carcinogens [6].

Helminths have a complex biological cycle that includes various host organisms in which they experience multiple developmental stages and metabolic adaptations [7,8]. They can be transmitted in a variety of ways; consequently, they have several routes of invasion [9]. However, they all have in common the amazing ability to modulate the host’s immune response, suppressing responses that help its elimination and resolution of the infection [10]. This immunomodulatory capacity is related to the release of excretory–secretory (ES) products, such as metabolites, proteins, lipids, and extracellular vesicles (EVs), mediating host–parasite interaction [10,11,12]. EVs loaded with small non-coding RNAs (ncRNAs) are considered an important cross-species communication mechanism and represent a potential therapy for some infectious diseases [13].

MiRNAs are a group of ncRNAs (about 18–25 nucleotides) that regulate gene expression at the post-transcriptional level through their interaction with the 3′ untranslated regions of target mRNAs, promoting translational inhibition or mRNA destabilization and degradation. In addition, they regulate various biological processes, such as cell death, signal transduction, development, homeostasis, cellular proliferation, and differentiation [14,15]. Thus, miRNAs play an important role during host–pathogen interactions, modulating many biological aspects in the pathogen and host cells [15,16,17]. For instance, miRNAs delivered by host cells and pathogens through EVs are critical mediators of cell–cell communication [18,19]. On the other hand, in parasitic amoebas, miRNAs regulate the expression of proteins involved in nutrient capture, signal transduction, cell motility, and polarization; in the infected cell, they are associated with the regulation of cell cycle arrest and apoptosis [18,20,21]. Since miRNA expression changes during pathogen infection, they have been proposed as a tool for the prevention, diagnosis, prognosis, treatment, and control of parasitic diseases [16,22,23,24]. In addition, several miRNAs have been suggested as biomarkers of some parasitic diseases’ different phases and severity [24,25,26].

This review focuses on analyzing the possible role of miRNAs in infections caused by parasitic helminths *Schistosoma, Fasciola,* and *Brugia*, with particular emphasis on their contribution to host immune modulation.

## 2. MiRNAs as Possible Diagnosis, Prognosis, Prevention, and Treatment Tools

Since miRNAs play a relevant role in the development and progression of human diseases, they have been proposed as tools to detect and predict neurodegenerative diseases, infections, and cancer. In all of them, the expression levels of specific miRNAs are altered [27,28]. Moreover, variations in the expression of miRNAs could also serve as a tool to predict changes in physiological processes [29]. Thus, changes in miRNA expression in pathologies are detectable in biological fluids and can be tissue-specific [30,31,32]. Moreover, miRNAs can be modulated at the level of biogenesis or by adjusting their mode of action. Strategies for intervening biogenesis include using small-molecule drugs, miRNA sponges, and oligonucleotide therapies (miRNA replacement, antisense oligonucleotides). In addition, different miRNA delivery systems are currently being studied and developed, such as biomimetic delivery systems and synthetic nanoparticles. Additionally, miRNAs can be administrated in vivo and present an apparent lack of adverse events when administered intravenously [31,33,34].

Regarding the use of miRNAs as prevention tools, particularly in malaria, miRNAs are associated with the different phases of the infection and might allow the identification of carrier vectors and infected patients, symptomatic or not [23]. In addition, miRNAs could also be a tool to prevent the congenital transmission of parasitic diseases. For instance, miRNAs codified at the C19MC cluster are placenta-specific [31] and are differentially expressed in response to *Trypanosoma cruzi* and *Toxoplasma gondii* infection [35,36]. Moreover, some of these placenta-specific miRNAs can be detected in blood plasma or exovesicles [37] and might serve as prognostic markers. They could even serve as a strategy to assess the probability of vertical transmission. This would allow timely treatment for both the mother and the newborn.

## 3. Helminths and Neglected Tropical Diseases

Diseases caused by helminths affect 25% of the world’s population, especially in countries with lower resources and poor sanitation systems located in tropical and subtropical zones, mainly in Latin America, sub-Saharan Africa, China, and East Asia [10,38]. Therefore, these infections are considered a major public health problem worldwide due to their economic, social, and health impact [39,40,41]. In addition, these helminthic diseases are co-endemic with other infectious diseases, such as malaria and HIV/AIDS, causing an exacerbated progression [3].

**Schistosomiasis**, also known as bilharzia, is caused by six species of trematodes genus Schistosoma, where *S. mansoni*, *S. japonicum*, and *S. heamatobium* are the most prevalent [42]. Thus, urogenital schistosomiasis is caused by *S. haematobium*, whereas *S. mansoni* and *S. japonicum* are responsible for intestinal schistosomiasis. Furthermore, the advanced forms of schistosomiases lead to insufficiencies in other organs, bladder cancer, and ectopic pregnancies due to an increased susceptibility to other infections [43,44].

An estimated 236.6 million people are infected worldwide, more than 90% of whom live in Africa, and 200,000 people die from it yearly [45]. This disease mainly affects fishing and agricultural populations, where women with domestic work and school-aged children are the most vulnerable group [46]. The annual economic losses associated with schistosomiases exceed USD 600 million [47].

**Fascioliasis** is a zoonotic disease transmitted by ingesting contaminated aquatic plants, mainly affecting populations of South America, Southeast Asia, Africa, and Hawaii [48,49]. This disease is caused by the flukes *F. hepatica* and *Fasciola gigantica* (*F. gigantica*); they infect many mammals, including livestock and humans, causing short- and long-term complications [50]. Children are the most vulnerable to these complications, which include malnutrition, anemia, stunted growth, and cognitive retardation [51]. It is estimated that 50 million people are infected worldwide, and 180 million are at risk of contracting the disease [52]. The economic impact of this disease on the livestock industry is mainly associated with the loss of animal productivity, export restrictions, and reduced consumer demand. In the North and South American livestock industry alone, fascioliasis contributes to losses of over 2 billion dollars per annum [53].

**Lymphatic filariasis**, commonly known as elephantiasis, is a devastating and disabling disease that affects the lymphatic system, causing pain, hypertrophy of some parts of the body, and social stigma. Three species of the spiuririd nematodes cause this disease: *Brugia malayi*, *B. timori*, and *Wuchereria bancrofti* (*W. bancrofti*) [54]. This infection affects more than 120 million people worldwide, and is the second most common cause of long-term disability, surpassed only by mental illness [55,56,57]. Depending on the species causing the filariasis, the host immune response, and secondary infections, the individual may be asymptomatic or present a broad spectrum of symptoms. The symptoms include lymphedema, hydrocele, chyluria, chylous diarrhea, and chylorrhagia [58,59]. Annually, this disease causes an economic burden of nearly USD 5.8 billion, attributed primarily to people with an advanced-stage disease who cannot engage in normal productive activities [60].

## 4. Modulation of Host Immunity by Helminth Parasites

Although helminths show significant differences in their life cycle and tissue tropism, these parasites have in common that during infection, they carry out different modulatory strategies that affect all phases of the host’s immune response, establishing the infection. A wide variety of ES products mediates their ability to manipulate host immunity [61,62] during acute and chronic helminth infection [10]. Although these ES products differ between species, they have a common mechanism of action, the simultaneous promotion of regulatory and proinflammatory Th2 immune responses. This response is also known as a modified Th2 response through pattern recognition receptors (PRRs) such as Toll-like (TLRs) receptors [9].

EVs are particularly important ES since they can transport miRNAs that regulate the host’s gene expression after its internalization. Not surprisingly, most of the genes regulated by these EVs are involved in biological processes and pathways associated with pathogenicity and the host immune response [63,64,65].

On the other hand, miRNAs present regulatory functions associated with growth, development, and response to drugs in the helminths [66,67,68,69,70]. In some helminth parasites, miRNAs constitute more than 70% of the ncRNAs in EVs [65,71].

During infection, dysregulation of miRNAs is related to the pathogenesis of diseases. Thus, host and parasite miRNAs determine the probability, progression, and establishment of the disease; consequently, they are considered master regulators of host–parasite interactions [15].

In the following section, we analyze the possible role of miRNAs in infections caused by helminth parasites, emphasizing their contribution to the immunomodulatory capacity of helminths during pathogen–host interactions.

## 5. miRNA in Schistosoma–Host Interaction

**Figure 1:**
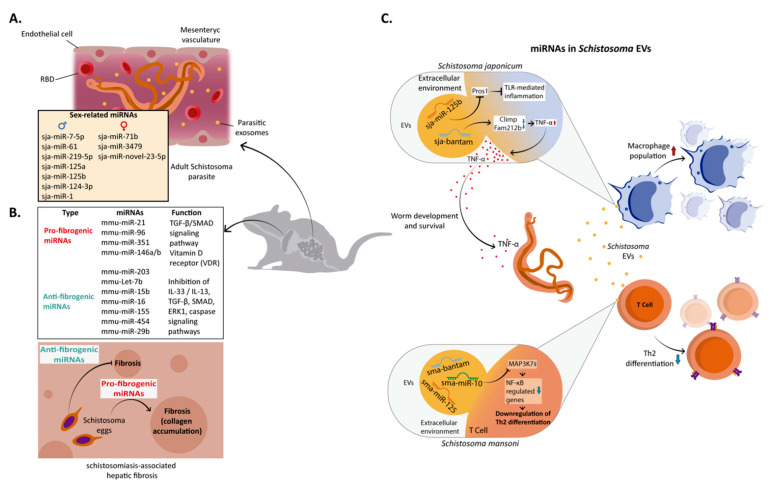
microRNAs during *Schistosoma* infection. (**A**) MiRNA expression in *Schistosoma.* In the parasite, the expression of miRNAs varies at different developmental stages; some are gender-biased. Parasite miRNAs are involved in the regulation of sexual differentiation, maturation, mating, and reproduction; (**B**) Role of miRNAs in liver injury and hepatic fibrosis. Several miRNAs are dysregulated and documented as pro-fibrogenic (favoring fibrosis; red) or anti-fibrogenic (inhibiting fibrosis; green). *Schistosoma* eggs produce both responses in liver tissue. Adult *Schistosoma* parasites accumulate in mesenteric vasculature and release miRNAs-EVs with immunomodulatory effects; (**C**) Effects of *Schistosoma* miRNAs released in EVs on target cells. *Schistosoma* releases miRNAs-EVs to regulate the host’s immune response. These miRNAs-EVs are composed of sma-miR-10, sma-miR-125, and sma-bantam in *S. mansoni*, while miR-125b and bantam are found in *S. japonicum* EVs. During *S. mansoni* infection, EVs fuse with T lymphocytes. The release of miR-10 into the T cell cytoplasm modulates the signaling pathway through MAP3K7 and regulates genes negatively depending on NF-kβ activation, diminishing T cell differentiation into Th2 subpopulations. Alternatively, during infection of mice by *S. japonicum*, EVs fuse with macrophages, and miRNA content inhibits the TLR-mediated inflammation (Sj-miR-125b) or stimulates the production and release of TNF-α to the extracellular environment, a molecule related to parasite development and survival. This process also promotes the increase in the macrophage population and may influence gene expression in *Schistosoma*. Adobe Illustrator was used to elaborate the figure.

*Schistosoma*’s miRNAs play a relevant role during the establishment of infection [72,73,74] (Figure 1).

In these parasites, miRNAs represent 30% of the non-coding RNAs and can be located on both sex and autosomal chromosomes [75,76]. To date, 79 and 225 mature miRNAs have been reported in *S. japonicum* and *S. mansoni* (miRbase (Version 21), respectively, 12 of which are specific to the genus *Schistosoma* [74]. Interestingly, the miRNA profile varies at different stages of development and gender, suggesting that these molecules are relevant in morphogenesis, development, and reproduction [72,73,74]. Thus, various miRNAs exhibit sex-biased expression [74,77] (Figure 1A). In *S. japonicum*, sja-miR-7-5p, sja-miR-61, sja-miR-219-5p, sja-miR-125a, sja-miR-125b, sja-miR-124-3p, and sja-miR-1 are more abundant in male worms, while sja-miR-71b-5p, sja-miR-3479-3p, and sja-miR-novel-23-5p are expressed mostly in female worms [77]. The number of sex-biased miRNAs varies between *Schistosoma* species, being higher in *S. japonicum* than in *S. mansoni* [74,75]. Notably, the sex-biased miR-71/miR-2 cluster is highly conserved in other pathogenic flatworms, experienced a duplication process *in S. mansoni*, and is involved in regulating at least 389 genes [74,76]. The enrichment of these miRNAs may be evidence of their association with regulating cellular processes, including metabolism, glycosylation, cell cycle, genome stability, and DNA synthesis [78]. Many genes involved in these processes have target sites for some sex-biased miRNAs [74,77]. Thus, sja-let-7, sja-miR-1, sja-miR-7-5p, sja-miR-3479, sja-miR-71, sja-miR-71b-5p, and miR-71/miR-2 clusters have the most putative sites within sex-biased genes [78]. In the context of gene expression influenced by gender, these specific miRNAs could serve as a candidate for the design of new vaccines against parasite fertility [78,79].

Regarding developmental stage regulation, different groups of miRNA are associated with each one, fulfilling regulatory functions [77,80]. For instance, in *S. japonicum,* 28 miRNAs are highly expressed in one or more developmental stages [77]. However, a set of miRNAs, including sja-miR-1, sja-bantam, sja-miR-124-3p, sja-miR-2a-3p, sja-miR-3492, and sja-miR-36-3p, are significantly suppressed in lung-stage schistosomula. Others, such as sja-miR-71, sja-miR-1, sja-miR-71b-5p, sja-miR-36-3p, and sja-miR-124-3p, have been the most abundant members at the egg stage [81].

In schistosomiasis-associated hepatic fibrosis, host miRNAs also have been linked with anti- and pro-fibrogenic roles [82] (Figure 1B). Liver fibrosis is caused by *Schistosoma* eggs that induce the formation of granulomas and the subsequent excessive deposition of extracellular matrix (ECM), including collagen fibers [83]. Thus, mmu-miR-21-5p, mmu-miR-96-5p, mmu-miR-351-3p, and mmu-miR-146 a/b activate different signaling pathways to promote schistosomiasis-associated hepatic fibrosis [82,84,85]. For instance, mmu-miR-21-5p and mmu-miR-96-5p activate the (TGF-β1)/SMAD signaling pathway, leading to an increase in IL-13 and TGF-β1, both associated with fibrosis. Moreover, this signaling pathway promotes the expression of mmu-miR-21-5p and mmu-miR-96-5p, and once overexpressed, both miRNAs inhibit the expression of their target SMAD7, leading to an increase in collagen expression. During the initial phase of Schistosomiasis, IFN-γ negatively regulates mmu-miR-351-3p in hepatic stellate cells (HSCs), facilitating the expressions of vitamin D receptor (VDR) and SMAD7 TGF-β/SMAD signaling antagonists and blocking the activation of HSCs. However, as egg deposition occurs in the liver, secreted cytokines switch from the Th1-type to the Th2-type, and consequently, IFN-γ concentration decreases, mmu-miR-351 increases, HSC is activated, and collagen (Col1α1, Col3α1) is produced [82,84,85].

Additionally, miR-146 a/b members regulate the differentiation of macrophages into M2 cells [86], which attenuate excessive inflammatory processes. Furthermore, they promote protective responses of the host by secreting cytokines such as IL-10 and TGF-β [87]. IL-10 presents immunosuppressive roles in helminthic infections, and TGF-β promotes tissue fibrosis through the overproduction of type I collagen [88,89].

Anti-fibrogenic roles of miRNAs in schistosomiasis have also been documented [90,91,92,93] (Figure 1B). Thus, mmu-miR-203-3p, mmu-let-7b-5p, mmu-miR-15b-5p, mmu-miR-16-5p, mmu-miR-155-5p, mm-miR-454-3p, and mmu-miR-29b-3p regulate the fibrogenic process through the inhibition of different signaling pathways, including the IL-33/IL-13, TGF-β, SMAD, ERK1, PI3K/AKT, and caspase ones. In addition, these miRNAs target some proteins involved in these pathways, such as Smad3, TβRI, FOXO3a, Bcl2, COL1A1, and Col3a1 [82,90,91,92,93]. It has been proposed that the decrease in cytokine IL-33 secretion, an inducer of type 2 immunity, is a mechanism by which mmu-miR-203-3p could inhibit *Schistosoma* infection-associated liver fibrosis [94]. Notably, the regulation of TLR2 expression by mmu-miR-92a-2-5p also inhibits *S. japonicum*-induced liver fibrosis. Although the mechanism for this inhibition of fibrosis is unclear, it has been postulated that it could occur through the promotion of apoptosis of fibroblasts [95].

TGF-β is critical in the development and male–female interactions of *Schistosoma* as well as during host–parasite interaction [96]. TGF-β signaling pathways are present in *S. mansoni,* and they are activated by the binding of human ligand TGF-β1 to TGF-β type II receptor (SmTβRII) exposed on the tegument of the parasites. The consequent activation and nuclear translocation of the SMAD multiprotein complex promote the transcription of target genes involved in parasite development and host–parasite interaction [96,97].

In the spleen and lungs, *Schistosoma* infection also upregulates different miRNAs related mainly to immune response, nutrient metabolism, cell differentiation, apoptosis, and signal pathways [73]. For example, MAPK, insulin, TLRs, and TGF-β signaling pathways are those mainly regulated by differentially expressed miRNAs in response to *S. japonicum* [98].

In addition to regulating some cellular processes in the parasite, the helminth miRNA-EVs modulate host gene expression and facilitate the dissemination of the pathogen [99,100] (Figure 1C).

For instance, *S. japonicum* miRNAs released in EVs regulate host macrophage functions, facilitating parasitism. Uptake of sja-miR-125b and sja-bantam increases macrophage proliferation and TNF-α production by repressing miRNA targets, including Pros1, Fam212b, and Clmp [101] (Figure 1C). Sja-miR-125b targets the Pros1 proteins, which are known to inhibit the TLR-triggered inflammatory responses. In the case of sja-bantam, it regulates the expression Clmp and Fam212b to affect TNF-α production. Additionally, sj-miR-125b and bantam influence macrophage proliferation. These results suggest that sj-miR-125b and sja-bantam can regulate TNF-α production by reducing levels of Pros1, Fam212b, and Clmp and altering macrophage function. Moreover, TNF-α has an autocrine effect during macrophage differentiation and positively influences parasite development, metabolism, and egg laying of *Schistosoma* [102,103,104].

In addition, host TNF-α can induce differential gene expression and protein phosphorylation in schistosomes [105,106] due to TNF-α receptors in *S. mansoni* [105]. Therefore, the increased proliferation of TNF-producing cells could be a mechanism by which these parasites modulate gene expression in the host [104,105].

In chronic *S. mansoni* infection, sma-miR-10-5p, sma-miR-125b, and sma-bantam are released in EVs by adult parasites. These miRNAs modulate host T helper cell differentiation. Thus, miRNA-EVs are incorporated by Th cells, where the miRNAs downregulate the Th2-specific transcriptional program [100]. The Th2 pathway is a significant player in response against the helminth parasites and other extracellular parasites [107,108]. Mainly, sma-miR-10-5p is responsible for downregulating the expression of genes under the control of NF-*kB*, a transcription factor essential for Th2 differentiation, and represses the serine/threonine kinase MAP3K7 expression in the presence of *Schistosoma* parasites [100] (Figure 1C).

*Schistosoma*-derived miRNAs can be detected in the serum/plasma of the host [109,110,111]. Particularly, sma-miR-277, sma-miR-3479-3p, and sma-bantam have been detected in the serum of *S. mansoni*-infected mice and patients [109]. BALB/c mice infected with *S. japonicum* evidenced the presence of 21 parasite-derived miRNAs (sja-miR-277, sja-miR-3479-3p, sja-miR-125a, sja-miR-61, sja-miR-2162-3p, sja-miR-36-3p, sja-miR-3489, sja-miR-3487, sja-miR-10-5p, and members of the miR-2 cluster (sja-miR-2a/2b/2c). Interestingly, six of these miRNAs (sja-miR-277, sja-miR-125a, sja-miR-36-3p, sja-miR-2a, sja-miR-2b, and sja-miR-2c) were also identified in infected human serum [110]. Notably, sja-miR-2b-5p and sja-miR-2c-5p can be detected infected individuals with a low parasite load. Others, such as sja-miR-2c-5p, sja-miR-277, and sja-miR-3479, significantly correlate with fecal egg counts and hepatic egg burden [110,112]. Therefore, it has been proposed that these parasite-derived circulating miRNAs could serve as tissue or serum biomarkers for detecting human *S. japonicum* infection, even in low-intensity infections [109,110,111].

In some host tissues, such as the liver, spleen, and lungs, *Schistosoma* induces altered expression between 130 and 220 miRNAs [73,98,113]. Some of those miRNAs (mmu-miR-146b, mmu-miR-155, mmu-miR-223, mmu-miR-142-3p, mmu-miR-15b, mmu-miR-126-5p, mmu-miR-199a-5p, mmu-miR-134, and mmu-miR-214) increase their expression significantly at different times post-infection [73,98]. In infected liver cells, highly elevated expressions of mm-miR-34c, mmu-miR-134, mmu-miR-223, mmu-miR-199a-5p, and mmu-miR-214 have been reported [73], suggesting their role in hepatic disease [113].

Therefore, in schistosomiasis, differential expression of miRNAs is observed in the host and in the parasite itself. In parasites, these miRNAs are involved in different aspects of the parasite’s biology and are part of a manipulation mechanism of the host’s immune system. In the host, in addition to having regulatory roles in infection, it influences the development of the parasite and hepatic disease.

## 6. miRNA in Fasciola–Host Interaction

**Figure 2:**
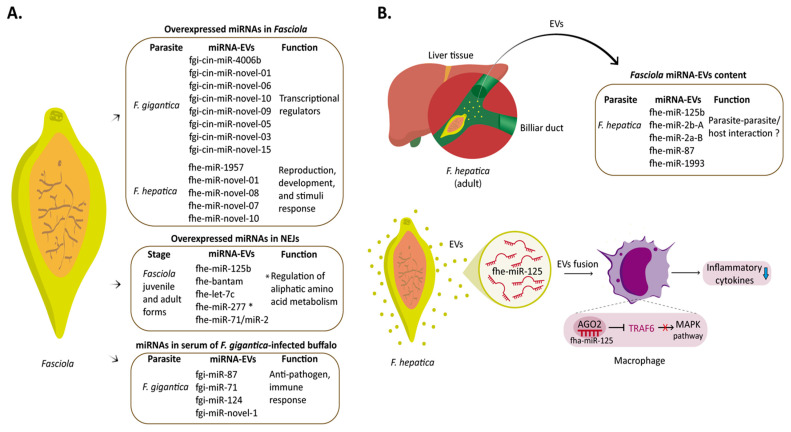
microRNAs during Fasciola infection. (**A**). miRNAs are expressed differentially in the morphological stages of Fasciola. Thus, different miRNAs are expressed in adult parasites and juvenile stages of *F. gigantica*. (**B**). *F. hepatica* releases miRNAs in EVs involved in parasite–host interaction. For example, *F. hepatica* releases fhe-miR-125, which hijacks the host macrophage miRNA machinery mimicking host miR-125b to regulate innate immune responses. Adobe Illustrator was used to elaborate the figure.

Fasciola parasites are macroscopic organisms living in a hostile environment, such as the mammalian liver and bile ducts, and possess sophisticated mechanisms to evade host immunity. MiRNAs play a crucial role in the development and pathogenesis of this disease [114,115] (Figure 2).

Like other trematodes, Fasciola produces its miRNAs [21]. At the adult stage, *F. gigantica* and *F. hepatica* present 19 and 16 miRNA candidates, respectively, and share 11. Another eight miRNAs, fgi-cin-miR-4006b, fgi-cin-miR-novel-01, fgi-cin-miR-novel-06, fgi-cin-miR-novel-10, fgi-cin-miR-novel-09, fgi-cin-miR-novel-05, fgi-cin-miR-novel-03, and fgi-cin-miR-novel-15, are specific for *F. gigantica,* and five are specific for *F. hepatica,* namely fhe-mmu-miR-1957, fhe-miR-novel-01, fhe-miR-novel-08, fhe-miR-novel-07, and fhe-miR-novel-10 (Figure 2A). Interestingly, eight miRNAs are conserved to the parasites *S. japonicum* and *S. mansoni* [21]. For instance, sja-bantam, sja-let-7, sja-miR-10 and sja-miR-125a, and members of mir-71/miR-2 clusters (sja-miR-71, sja-miR-2b/2e) are shared with *S. japonicum* [80]. These miRNAs are associated with sex determination, regulation of developmental stages, and dissemination of the pathogen [74,77,81,100]. As related organisms, it is likely that they have similar metabolic processes and share similar miRNAs.

Notably, the target genes of Fasciola species miRNAs differ in *F. gigantica* and *F. hepatica* (Figure 2A). In the case of *F. gigantica*, the predicted targets were mostly transcriptional regulators. In contrast, for *F. hepatica,* the predicted targets are proteins related to reproduction, development processes, response to stimuli, immunomodulation, and locomotion, suggesting different mechanisms of gene regulation between the two parasites [21]. These differences can be attributed to the intermediate hosts, morphological characteristics, geographic distribution, and metabolic adaptations during their life cycle in both species [116,117]. Differences in gene target prediction results could also influence the observed result [118].

In the encysted juvenile stage (NEJs) of *F. hepatica*, miRNAs play an important role in the invasion process [119]. Moreover, at this stage of development, miRNAs previously reported in the adult stage are also highly expressed (fhe-miR-125b, fhe-miR-bantam, fhe-let-7c, fhe-miR-277, and fhe-miR-71/miR-2 cluster members) [21]. Notably, miR-277 is related to the regulation of enzymes involved in the catabolism of aliphatic amino acids [120], having an essential role in the survival of the parasite under conditions of stress or starvation. These miRNAs are also associated with specific gene regulation expression needs in NEJs [119].

Like other flatworms, *F. hepatica* can secrete EVs that contain miRNAs [121,122,123] (Figure 2B). Thus, in *F. hepatica*, 54 miRNAs have been isolated from EV, being fhe-miR-125b, fhe-miR-2b-A, fhe-miR-2a-B, fhe-miR-87, and fhe-miR-1993 among the most highly expressed [122]. Notably, fhe-miR-125b hijacks host macrophage miRNA machinery and modulates early innate immune responses. When released by *F. hepatica* and internalized by macrophages, fhe-miR-125b loads onto the mammalian Argonaut protein (Ago-2), mimicking host miR-125b and decreasing inflammatory cytokines by interrupting the MAPK signaling pathway through TRAF6 targeting [123] (Figure 2B).

In humans, the hsa-miR-125 family is crucial during immune system development, immune modulation, tumor promotion and suppression, and host–pathogen interactions (Su et al., 2013). On the other hand, fhe-miR-87 and fhe-miR-1993 are absent in mammals. However, fhe-miR-2b-A and fhe-miR-2a-B are orthologs of miR-27, bta-mir-27a, and bta-mir-27b [122]. Therefore, these miRNAs-EVs may have an important role in parasite–parasite and host–parasite communication processes [124].

The host’s circulating miRNA profile is dysregulated during Fasciola infections [125]. For example, in *F. gigantica*-infected ruminants, a differential expression of 121 circulating miRNAs was observed (44 miRNAs were upregulated, and 77 miRNAs were downregulated). The target genes of these differentially expressed miRNAs are related to the regulation of signal transduction, immune response, and organelle localization. Moreover, four parasite-derived miRNAs (fgi-miR-87, fgi-miR-71, fgi-miR-124, and fgi-miR-novel-1) were detected in the serum of *F. gigantica*-infected buffalo [125]. Of these, miR-87 and miR-71 have been associated with anti-pathogen and immune responses [126].

Therefore, during Fasciola infections, changes in miRNA expression occur in the host and parasite. Like *Schistosoma*, these are key players in the parasite’s biology and manipulation of the host’s immune responses. In addition, some of these are unique to this parasite, making them targets for possible therapeutic strategies.

## 7. miRNA in *Brugia malayi*–Host Interaction

Filarial parasites employ several strategies to evade the immune response during infection. Most of these strategies are orchestrated by ES, which interferes with the functions of the host’s intracellular and extracellular immune machinery [127]. Thus, parasite miRNAs have relevant roles in parasite biology and immune dysfunction in the host [128,129].

**Figure 3:**
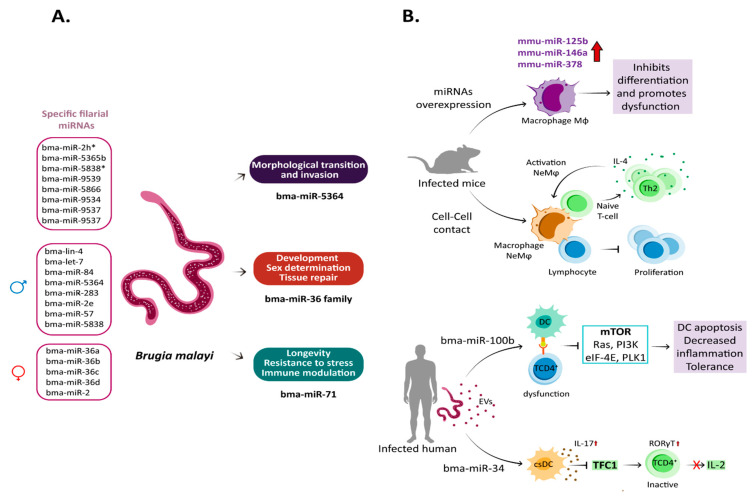
microRNAs during *B. malayi* infection. (**A**). MiRNAs identified in *B. malayi*. Specific miRNAs with gender-biased expression have been identified in *B. malayi*. Thus, bma-miR-5364, bma-miR-71, and the bma-miR-36 family regulate biological processes, including host immune modulation. (**B**). Alteration of host’s miRNAs during *B. malayi* infection. Infection by this parasite induces miRNA upregulation related to immune dysfunction. Alternatively, this parasite can induce NeMφ differentiation that can exert an anti-proliferative effect on lymphocytes, mediated by cell-to-cell contact. For its part, miRNA EVs derived from the parasite (bma-miR-100 and bma-miR-34) are internalized by functional DCs. In the cytoplasm, they modulate mTOR signaling pathways, cellular proliferation, differentiation, and apoptosis-related genes. Adobe Illustrator was used to elaborate the figure.

In *B. malayi*, 145 miRNAs have been identified. They are grouped into 99 families, of which 61 are highly conserved with homologs in arthropods, vertebrates, and helminths, and nine appear to be filaria-specific. Several miRNA families differ depending on the development stage and gender [128] (Figure 3).

Within these filaria-specific miRNAs are included bma-miR-2h*, bma-miR-5365b, bma-miR-5838*6, bma-miR-9539, bma-miR-5866, bma-miR-9534, bma-miR-9537, and bma-miR-9537 [128]. Notably, bma_miR-5866 is one of the most abundant in this parasite and has been identified in other helminth species, including *B. pahangi* and *W. bancrofti*. Bma-miR-5866 is very similar to the miR-57 family of *B. malayi* and *C. elegans*, suggesting that it acts as a regulator of embryogenesis and larval development [128,130]. MiR-9536 is another interesting filaria-specific miRNA since it is in an intron of Bm1_03065, a cut-1 cuticlin gene fragment [128]. Cuticlin is an insoluble protein component of the nematode cuticle, regulated post-transcriptionally in other helminths [131,132]. Thus, miR-9536 expression is likely coordinated with genes involved in cuticle molting or synthesis [128].

Like other helminths, *B. malayi* presents various miRNA with gender-biased expression (Figure 3A). Thus, the bma-let-7 family (bma-lin-4, let-7, bma-miR-84, bma-miR-5364), bma-miR-283, bma-miR-2e, bma-miR-57, and bam-miR-5838 are more abundant in male parasites, while bma-miR-2b-2* and the bma-miR-36 family (bma-miR-36a/bma-miR-36b/bma-miR-36c/bma-miR-36d) have a higher expression in female parasites [128]. In *B. pahangi*, miR-84 and let-7 are also abundant in males [133]. Although the implications of gender-based differential expression of these miRNAs have been poorly studied, they most likely regulate the 1930 genes with sex-based expression in *B. malayi*. Protein kinases, phosphatases, and sperm proteins are preferentially transcribed genes in adult males. Transcription factors, nuclear receptors, serpin activity, and structural constituents of the cuticle (such as collagen) are among the genes highly transcribed by adult females [134].

Let-7 and miR-2b are key players in expressing some gender-associated genes in *S. japonicum* [78]. In *C. elegans,* lin-4, let-7, and miR-84 regulate the temporal events of development and larval-to-adult transition. In this transition, let-7, lin-4, and miR-84 together regulate genes of heterochronous pathways such as the transcription factor HBL-1, the orphan nuclear receptor DAF-12, and the nuclear protein Lin-41 [135,136]. In *B. malayi* males, these miRNAs also synchronize sex determination pathways, similar to other helminths [137].

Bma-miR-5364 was described as essential an miRNA for morphological transition and host invasion by *B. malayi*, through the regulation of at least 13 genes, including zinc finger proteins, Ets domain-containing protein, LEM domain-containing protein, and high mobility group protein (HMG) [133], involved in sexual differentiation, transcriptional repression, regulation of DNA-dependent processes, apoptosis, and cell senescence [138,139,140,141]. Notably, bma-miR-5364 is specific to the species *Brugia* [128,133], *Ascaris suum* [142], and clade III parasitic nematodes [143], making it an attractive therapeutic target.

For its part, members of the miR-36 family are mainly expressed in female adults and embryogenic stages of *B. malayi* [143] (Figure 3A). This family of miRNA appears to be helminth-specific, and its absence in some nematodes is lethal [144,145]. It has been identified both in parasitic worms and free-living ones, and its functions are related to development, sex determination, and tissue regeneration [74,146,147,148,149].

Like *S. mansoni*, miR-36a has gender-biased expression in *B. malayi* adult females [74]; in free-living flatworms such as planarian *Schmidtea mediterranea*, sme-mirR-36B expresses specifically in neoblasts [146,148]. In addition to sme-miR-36a, sme-let-7a and sme-miR-2a are also expressed in neoblasts, regulating the expression of genes involved in the regenerative capacity and differentiation of stem cells [150,151].

On the other hand, bma-miR-71, bma-miR-92, bma-miR-153, bma-miR-2a, bma-miR-5366*, and bma-miR-5842* are highly expressed in the juvenile microfilariae (Mf) stage, while several members of the miR-2 (bma-miR-2b, bma-miR-2b-2*, bma-miR-2e, bma-miR-2h*, bma-miR-2i) and miR-36 (bma-miR-36a, bma-miR-36b, bma-miR-36c, bma-miR-36c/b*,bma-miR-36d and bma-miR-36d*) clusters are overexpressed in the adult state [128].

Notably, miR-71 is one of the most ubiquitous and conserved miRNAs in helminths, including *B. malayi,* and is associated with longevity, stress resistance, and neuron development [128,152,153] (Figure 3A). Thus, miR-71 modulates the expression of genes involved in the IGF-1/insulin-like pathway (AGE-1, PDK-1, AKT-1) and the genes CHK-1, CDC-25.1, and CDC-25.2, involved in DNA damage checkpoint pathways, which makes it a linker miRNA of both pathways [152]. Additionally, by regulating genes of the IGF-1/insulin-like pathway, miR-71 promotes the expression and activity of the forkhead transcription factor (DAF-16). DAF-16 is a FOXO family transcription factor that modulates antioxidant, antimicrobial, and metabolic enzymes necessary to extend the parasite’s lifespan [152,154]. The regulation of longevity by both signaling pathways allows Mf to circulate throughout the host body for a long time until a mosquito ingests it. In addition, the expression of ammonium transport protein and proteins involved in the Lin-12/Notch signaling pathway have also been proposed as targets of bma-miR-71 in *B. malayi* [155].

MiR-71 also plays a role in host–nematode interactions [156,157]. It is released by EVs and is internalized by immune cells, where it regulates the production of nitric oxide (NO) and the expression of components of RISC and host miRNAs linked to inflammation [157]. Additionally, it has been observed that EVs loaded with miR-71 from *Heligmosomoides polygyrus* and administered intranasally in BALB/c mice promote the suppression of type 2 cytokines (IL-5 and IL-13) in innate lymphoid cells, as well as the expression of the IL33-receptor and phosphatase DUSP1 in recipients cells [156]. Although DUSP1 regulates the inflammatory response, alterations in its expression dampen the acute inflammatory response by favoring macrophage’s arginase expression over nitric oxide synthase [158]. Moreover, reduced expression of DUSP1 results in GATA-2 phosphorylation and inhibition of its ability to promote IL33r transcription [159].

The function of the miR-2 cluster is less known in *B. malayi;* however, there have been associations with parasite embryogenesis [128]. In other helminths, the miR-2 cluster is involved in apoptosis suppression, development, and stress responses [66,143,145]. In *B. pahangi,* the miR-2 cluster members pba-miR-2a and pba-miR-2b are temporally expressed and are more abundant in adult worms in a similar way as miR-7 and miR-36 [143]. Interestingly, in *Schistosoma*, the miR-2 orthologue is clustered with miR-36 and participates in regulating sexual differentiation and maturation [78], evidencing that gene clusters can change the context of their function among helminths.

In mice, infection by *B. malayi* induces overexpression of mmu-miR-125b-5p, mmu-miR-146a-5p, and mmu-miR-378-3p in macrophages [160] (Figure 3B). These miRNAs activate macrophages, inflammatory response, and cell–cell communication [160,161,162]. For example, mmu-miR-125b-5p is involved in morphological changes during macrophage activation, increasing the expression of co-stimulatory molecules (such as CD80) and INF-γ secretion through the suppression of IFN regulatory factor 4 (IRF4) [161]. IRF4 is a transcription factor involved in the expression of several IFNs, proinflammatory cytokines, and chemokines; it is activated by activating TLR and the associated NF-kB pathway [163].

Infections by adult stages of *B. malayi* induce the production of IL-4 by the host, promoting the appearance of suppressive cells known as IL-4-dependent F4/80+ macrophages (or nematode elicited macrophages (NeMφ)), which exert an anti-proliferative effect on lymphocytes, mediated by cell-to-cell contact [164,165], and at the same time act as antigen-presenting cells (APC) to stimulate naïve T cells and induce their differentiation towards Th2 and IL-4-producing cells [166]. This facilitates the activation of suppressive NeMφ macrophages, originating a positive feedback loop that maintains an environment with abundant unresponsive immune cells that contributes to the establishment of infection (Figure 2B).

Alternatively, the induction of IL-4 production also would be a mechanism used by this parasite to induce some miRNAs and modulate the immune response. IL-4 induces overexpression of mmu-miR-378-3p in macrophages and suppression of proteins involved in the IL-4-receptor/PI3K/Akt-signaling pathway, exerting a negative regulation on the proliferation of macrophages [160]. It should be noted that the PI3K/Akt-signaling pathway directs various cellular processes in macrophages, so it is likely that other cellular processes are inhibited during the infection process [167] and lead to the immunosuppression seen in lymphatic filariasis [168] (Figure 3B).

In addition, *B. malayi* also releases miRNAs in EVs, modulating host responses [122,129,169]. At least 576 proteins and 130 miRNAs have been identified in *B. malayi* EVs. For instance, bma-miR-100, bma-miR-7, bma-miR-71, bma-let-7, bma-miR-99, bma-miR-9, bma-miR-34, bma-miR-31, bma-miR-92, bma-miR-55, and bma-miR-4299, have immunomodulatory properties by downregulating proteins involved in the mTOR pathway [129], an essential signaling pathway for immune regulation and cell proliferation and differentiation [170]. Thus, these filarial miRNAs target hosts’ mTOR, Ras, PI3K, eIF-4E, and PDK1 (Figure 3B). The alteration of this pathway might explain the antigen-presenting cell dysfunction observed in filarial infections [129]. Additionally, inhibiting the mTOR pathways should affect others involved in autophagy, glycolysis, pentose phosphate pathways, and de novo lipid synthesis [171].

Another explanation for the immunomodulating capacity of filarial miRNAs released in EVs is their homology to their human counterparts. For instance, bma-let-7, bma-miR-9, bma-miR-92, bma-miR-100b, and bma-miR-34 are 98–100% homolog to their human counterparts [169]. Particularly, bma-miR-100b and human has-miR-100 target mTOR and increase apoptosis by downregulating polo-like kinase 1 (PLK1) [172,173]. PLK1 is a proinflammatory cytokine; it is activated by TLR and is involved in T-cell apoptosis [174,175] (Figure 3B).

For its part, bma-miR-34 could also be vital in immunomodulation during filarial infections [129]. Bma-miR-34 overexpression decreases CXCL10/CXCL11/CXCR3 secretion in immune cells, impairing their migration and activation [176]. In addition, miR-34 overexpression of miR-34a promotes differentiation of a conventional dendritic cell (csDC), producing high amounts of IL-17, which leads to inhibition of CD4+ T cell activation through the repression of transcription factor T cell factor 1 (TCF1) and consequent increases in the orphan nuclear receptor RORγT expression [67]. The latter inhibits cytokine IL-2 secretion in T cells [177,178] (Figure 3B).

## 8. Conclusions

Helminth parasites are a globally distributed group of organisms with great clinical relevance, representing a risk for humans and animals. They have an amazing ability to modulate their host’s immune response through sophisticated strategies involving miRNAs. In the host, miRNAs modulate signaling pathways, resulting in an altered immune response favoring parasite persistence and tolerance. In the parasite, these molecules regulate their development and virulence. Therefore, studying miRNAs during host–parasite interaction is of great importance to improve prognostic, diagnostic, and therapeutic strategies for those neglected diseases caused by these parasites.
